# Laparoscopic management of type II Mirizzi syndrome

**DOI:** 10.1007/s00464-019-07316-6

**Published:** 2020-03-05

**Authors:** Fátima Senra, Lalin Navaratne, Asunción Acosta, Alberto Martínez-Isla

**Affiliations:** 1 Dept Surgery, St. Mark’s Hospital, Watford Road, London, HA1 3UJ UK; 2 Dept Surgery, Doctor Negrin University Hospital, Barranco de la Ballena, Las Palmas de Gran Canaria, 35010 Spain

**Keywords:** Transinfundibular approach, Type II Mirizzi, Mirizzi syndrome, Cholecystocholedochal fistula, Laparoscopy, LABEL

## Abstract

**Background:**

Mirizzi syndrome is an uncommon complication of longstanding gallstone disease. Pre-operative diagnosis is challenging, and to date, there is no consensus on the standard management for this condition. Until recently open cholecystectomy was the standard of care for type II Mirizzi syndrome (McSherry classification). The objective of this study was to assess the incidence and management of type II Mirizzi syndrome in patients with proven or suspected choledocholithiasis undergoing laparoscopic common bile duct (CBD) exploration and present our experience in the laparoscopic management of this rare condition over the last 21 years.

**Methods:**

Prospective data collection of eleven cases of type II Mirizzi syndrome amongst a series of 425 laparoscopic bile duct explorations was performed between 1998 and 2019. Demographic, clinical, diagnostic, intra-operative, and post-operative data were recorded.

**Results:**

The incidence of type II Mirizzi syndrome was 2.6% in 425 laparoscopic CBD explorations. All operations were completed laparoscopically with closure of the defect over a decompressed CBD (T-tube *n* = 3, antegrade stent *n* = 5, transcystic drain *n* = 2), and in one case a non-drained duct was closed with Endoloop. Stone clearance rate was 100% (11 cases). In two patients the transinfundibular approach was used in conjunction with holmium laser lithotripsy to enable choledochoscopy and successful stone clearance. Three patients were complicated in the post-operative period with bile leak (*n* = 2) and lower respiratory tract infection (*n* = 1). An incidental gallbladder carcinoma was found in one patient.

**Conclusion:**

Laparoscopic management of type II Mirizzi syndrome is feasible and safe when performed by experienced laparoscopic foregut surgeons. Laparoscopy and choledochoscopy can be combined with novel approaches and techniques to increase the likelihood of treatment success.

**Electronic supplementary material:**

The online version of this article (10.1007/s00464-019-07316-6) contains supplementary material, which is available to authorized users.

Mirizzi syndrome is an uncommon condition due to longstanding gallstone disease. It was first described by Kehr in 1905 and by Ruge in 1908 as a rare form of obstructive jaundice caused by external obstruction of the bile duct following impaction of a stone in the cystic duct with associated inflammation [1]. However, the condition was credited after Pablo Mirizzi in 1948, who defined it as compression of the hepatic duct by an impacted gallstone in the cystic duct or gallbladder neck [1–3]. This compression causes pressure ulceration that produces local inflammation. The compression and subsequent inflammation will lead to first external obstruction of the bile duct and further erosion into the bile duct, evolving into a cholecystocholedochal or cholecystohepatic fistula with different degrees of communication between the gallbladder and bile duct [4–8].

Since the description of its pathophysiology multiple classifications have been made, but McSherry’s and Csendes’ classifications are currently in wide use [1, 9]. McSherry proposed a classification of Mirizzi syndrome into two types based on ERCP findings. Type I is characterized by the extrinsic compression of the common hepatic or proximal common bile duct (CBD) due to an impacted gallstone in the infundibulum or cystic duct with subsequent inflammation, whereas type II is associated with a cholecystocholedochal fistula [4, 10]. Csendes’ classification further divides cholecystobiliary communication into three types according to the size of the cholecystocholedochal fistula in relation to the circumference of the CBD [1]. Its last modification in 2007 described a fifth type in which a bilioenteric fistula is present, with or without gallstone ileus [5, 7, 11]. McSherry’s classification was used in this study. The incidence of type II Mirizzi syndrome in all patients undergoing laparoscopic cholecystectomy from large national or regional databases is 0.04–0.08% [3, 12].

Pre-operative diagnosis of this condition is challenging, as there are no pathognomonic signs or symptoms. Obstructive jaundice and abdominal pain are the most frequent symptoms found in these patients [4, 10]. Diagnostic sensitivity of imaging tests is variable, ranging between 13% for ultrasound (US), 31% for computed tomography (CT), 76% for magnetic resonance cholangiopancreatography (MRCP), and 58% for endoscopic retrograde cholangiopancreatography (ERCP) [13]. Surgical management of Mirizzi syndrome is often dictated by the presence or absence of a cholecystobiliary communication. Laparoscopic cholecystectomy can be hazardous in Mirizzi syndrome due to the presence of severe local inflammation, fibrosis and adhesions within Calot’s triangle [3]. Current evidence still recommends open cholecystectomy for the management of Mirizzi syndrome [4, 12]. Some authors advocate a laparoscopic approach for type I Mirizzi syndrome only [7, 13, 14]. However, other authors recommend laparoscopic surgery [15–17] or combined pre-operative ERCP and laparoscopic subtotal cholecystectomy [18] for the treatment of type II Mirizzi syndrome.

The objective of this study was to assess the incidence and management of type II Mirizzi syndrome in patients with proven or suspected choledocholithiasis undergoing laparoscopic common bile duct exploration (LCBDE) and report our experience of the laparoscopic management of this rare and complex complication of gallstone disease over the last 21 years.

## Materials and methods

### Patients

A retrospective review of a prospectively maintained database of 425 consecutive patients who underwent LCBDE at a single centre between February 1998 and April 2019 was performed. Ethical approval was not required for this type of study. Based on McSherry classification, all type II Mirizzi syndrome patients were included for further analysis. All operations were performed or supervised by the senior surgeon (AI). All patients were assessed with pre-operative liver function tests (LFTs) and abdominal imaging: US, CT and/or MRCP. Four patients underwent pre-operative ERCP. Data collected included pre-operative demographic information and pre-morbid status, clinical presentation, pre-operative investigations, intra-operative findings (including use of intra-operative cholangiogram, IOC) and post-operative outcomes. Outcomes of this study were successful access to the CBD for choledochoscopy, successful stone clearance (if required), restoration of normal anatomy, conversion to open surgery, post-operative complications, and length of post-operative hospital stay. Normally distributed data are reported as mean (with standard deviation), whereas skewed or ordinal data are reported as medians. Categorical variables are expressed as number and frequencies (%).

### Surgical technique: transinfundibular approach

**Fig. 1:**
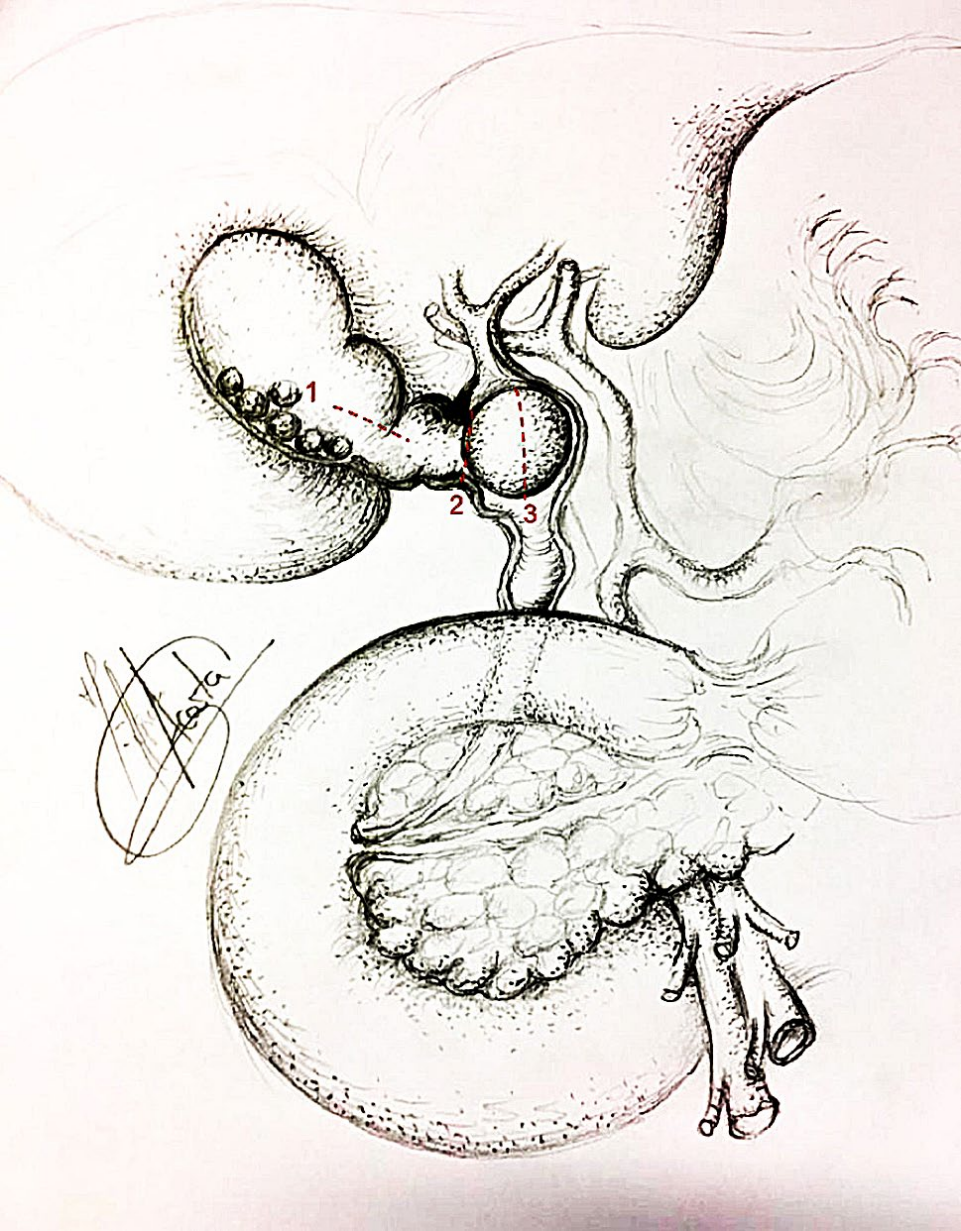
Incisions to access CBD in Mirizzi syndrome

**Fig. 2:**
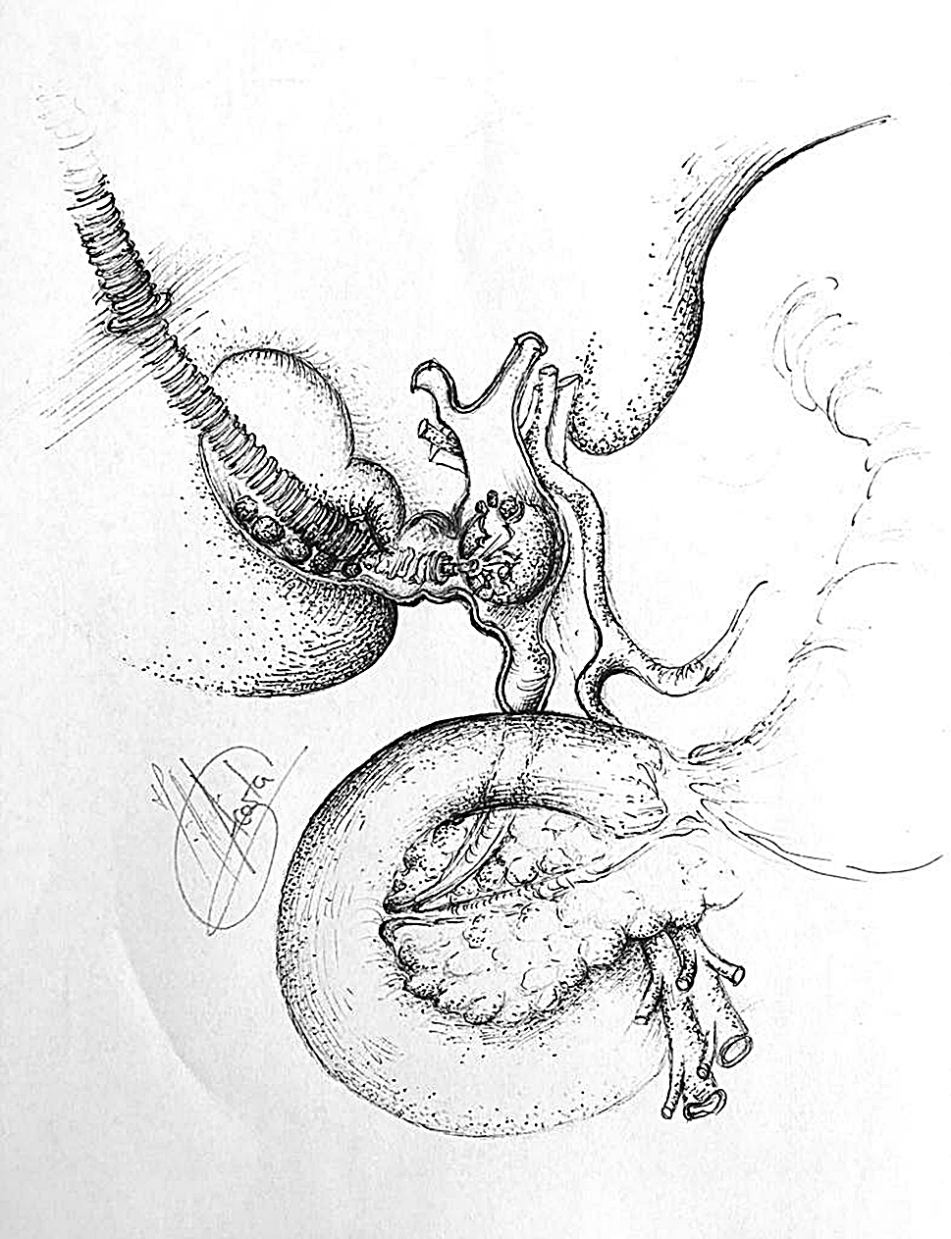
Stone duct clearance of CBD by LABEL

The French technique was used for positioning patients, with the surgeon standing between the legs and the assistant to the left side of the patient. A 5 mm 30° scope was sited through the abdominal wall at the level of the insertion of the ligamentum Teres to obtain an optimal view of the CBD and Calot’s triangle. A 5 mm Nathanson liver retractor was inserted in the epigastrium to maintain the operative view when necessary [19]. When no further dissection was possible within Calot’s triangle, hook diathermy (set to cutting) was used to create a longitudinal fistulotomy at the cholecystobiliary fistula. More recently in selected cases a longitudinal cholecystotomy at the gallbladder infundibulum was performed (transinfundibular approach, TIA) and the CBD subsequently accessed (Fig. 1) [19]. Stones impacted within Hartmann’s pouch were removed. In selected cases an IOC was performed using a cholangiocatheter (5-Fr ureteric catheter, open-end straight tip, 70 cm long, Cook Medical, Bloomington, IN, USA) through a Horner’s needle positioned in the right upper quadrant close to the costal margin. If transinfundibular intubation of the cystic duct was difficult, the cholangiocatheter was railroaded over a guidewire (PTFE Wire Guide with 3 cm flexible tip, 0.035 in diameter, 145 cm long, Cook Medical, Bloomington, IN, USA). Direct puncture of the gallbladder for IOC was not used as cholecystotomy was performed to access the CBD in cases with a frozen hilium (with or without pre-operative diagnosis). For cases within this series, choledochoscopy was performed through an extra 5 mm port in the right upper quadrant. Access to the CBD was achieved via the infundibulum or fistulotomy, and retrieval of stones (when required) was performed using standard retrieval techniques [19]. More recently (from February 2014), the laser-assisted bile duct exploration by laparoendoscopy (LABEL) technique, through the transinfundibular route was used in selected cases for fragmentation of large or impacted stones permitting complete CBD exploration and stone clearance whilst avoiding choledochotomy and/or fistulotomy (Fig. 2) [20, 21]. Closure of the CBD defect was mainly performed over a decompressed duct using a T-tube, antegrade stent or cholangiocatheter (10/11 patients). In one case with a moderately dilated infundibulum/cystic duct, closure was secured with an Endoloop (Ethicon, New Brunswick, New Jersey, USA). When external biliary drainage was used, a tubogram was performed 5–6 weeks after surgery to assess the anatomy and exclude bile leak prior to its removal. T-tubes were removed 6 weeks after surgery. Since its introduction in 2016, the transinfundibular approach has been used in cases where the hilum was frozen or inflamed, making laparoscopic exploration of the CBD a safe and feasible technique whilst avoiding the need for choledochotomy or fistulotomy (Fig. 1) [19, 22]. The LABEL technique was used as an adjunct in the management of large and/or impacted stones along the fistulous tract, allowing full access and subsequent complete clearance of the bile duct [5, 20, 22].

## Results

During the study period, 425 patients underwent LCBDE. Eleven patients were included after intra-operative findings fulfilled the diagnostic criteria for type II Mirizzi syndrome (McSherry classification).

The male to female ratio was 3:8 and the median age was 45 years old. Pre-operative assessment of fitness for surgery adopting the American Society of Anaesthesiologists (ASA) classification was as follows: ASA 1 (*n* = 4), ASA 2 (*n* = 5), and ASA 3 (*n* = 2). Pre-operative morbidity included hypertension (*n* = 5), hypercholesterolaemia (*n* = 3), diabetes mellitus (*n* = 2), ischaemic heart disease (*n* = 1), chronic pulmonary respiratory disease (*n* = 1) and asthma (*n* = 1). The most frequent clinical presentation was jaundice (*n* = 7). Two patients presented abnormal LFTs (with normal bilirubin) and another two patients with a dilated CBD on pre-operative imaging (with normal bilirubin and LFTs). Pre-operative studies included US (*n* = 9), CT (*n* = 4), MRCP (*n* = 6), and ERCP (*n* = 4) (Table 1). In three patients (27%) pre-operative imaging raised the suspicion of type II Mirizzi syndrome. In case 6, MRCP showed dilation and external compression of the CBD by a large stone in the cystic duct. In case 9, MRCP and CT reported a stone occupying the lumen of the gallbladder that eroded into the CBD suggestive of fistulisation. In case 11, MRCP showed images suggestive of communication between the gallbladder and the CBD. In our series the sensitivity for preoperative diagnosis of Mirizzi syndrome was 50% for MRCP, 25% for CT, and 0% for US. ERCP was performed in 4 patients. Clearance of the CBD by ERCP was not possible in the 4 cases due to difficult CBD cannulation (*n* = 1), inability to clear CBD stones (*n* = 1), inability to image and stent the CBD and perform sphincterotomy (*n* = 1), and inability to pass a guidewire due to bile duct stricture (*n* = 1). The sensitivity for preoperative diagnosis of Mirizzi syndrome by ERCP was 0% in our series. IOC was performed in four patients, with evidence of CBD stones in all cases, which were successfully removed with choledochoscopy and standard retrieval techniques.

**Table 1:** Preoperative data

Case number	Age	Gender	Clinical presentation	Bili (µmol/L)	Imaging
1	72	Female	Abnormal LFT	10	US
2	28	Female	Jaundice during pregnancy	28	CT
3	30	Female	Jaundice	30	US, CT, MRCP
4	45	Female	Jaundice	116	ERCP
5	81	Male	Dilated CBD	7	US, MRCP, ERCP
6	64	Female	Dilated CBD	8	US, MRCP^a^
7	30	Female	Jaundice	215	US, ERCP
8	72	Female	Jaundice	97	US, MRCP
9	73	Female	Abnormal LFT	8	US, CT, MRCP^a^, ERCP
10	38	Male	Jaundice	137	US, CT
11	26	Male	Jaundice	63	US, MRCP^a^

*Bili* Bilirrubin, *LFT* liver function tests, *CBD* common bile duct, *US* ultrasound, *CT* computed tomography, *MRCP* magnetic resonance cholangiopancreatography, *ERCP* endoscopic retrograde cholangiopancreatography

^a^Investigations that raised pre-operative suspicion of type II Mirizzi syndrome

**Fig. 3:**
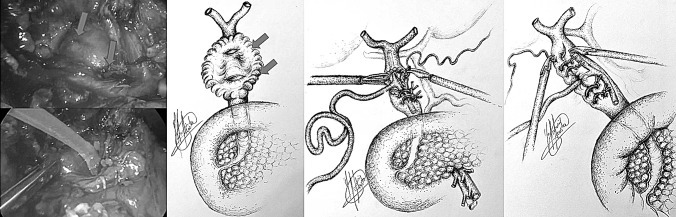
Large defect in CBD, closure over T-tube

All patients underwent laparoscopic subtotal/near total cholecystectomy after exploration of the CBD and extraction of any impacted stones through an incision at the site of the fistulous tract (fistulotomy) (*n* = 9) or infundibulum (TIA) (*n* = 2) (Table 2). In two patients transinfundibular Holmium laser lithotripsy (LABEL) was used to fragment and remove large impacted stones at the confluence between the cystic duct and CBD. CBD stones were present in 9 out of 11 patients and successful stone extraction was possible in all 9 patients (100%). In the other 2 patients the eroding stone was mainly lodged in the gallbladder and involved destruction of a large part of the CBD (Fig. 3).

**Table 2:** Intraoperative data

Case number	Findings	Diameter CBD (mm)	IOC	LABEL	Approach to CBD	Closure CBD	Intra-abdominal drain	Conversion to open surgery
1	Large stones in bile duct	25	No	No	Fistulotomy	T-tube	Yes	No
2	Small fistula with CBD, above stricture some stones	8	No	No	Fistulotomy	Stent	Yes	No
3	Morbidly obese. Hepatomegaly	9	Yes	No	Fistulotomy	Stent	Yes	No
4	Dilated short cystic duct, large Stone, small gallbladder	13	No	No	Fistulotomy	T-tube	Yes	No
5	Large stone in bile duct	15	No	No	Fistulotomy	Stent	Yes	No
6	Morbidly obese. Hepatomegaly	15	No	No	Fistulotomy	Stent	Yes	No
7	Large stone in bile duct. Low insertion of cystic duct	12	Yes	No	Fistulotomy	Transcystic drain	Yes	No
8	Acute cholecystitis	10	No	No	Fistulotomy	T-tube	Yes	No
9	Gallbladder cancer	12	No	No	Fistulotomy	Stent	Yes	No
10	Frozen hilium	12	Yes	Yes	Transinfundibular	Transcystic drain	Yes	No
11	Hiliar inflammation. Impacted stone CBD	10	Yes	Yes	Transinfundibular	Endoloop	No	No

*CBD* Common bile duct, *IOC* intraoperative cholangiogram, *LABEL* laser-assisted bile duct exploration by laparoendoscopy

**Fig. 4:**
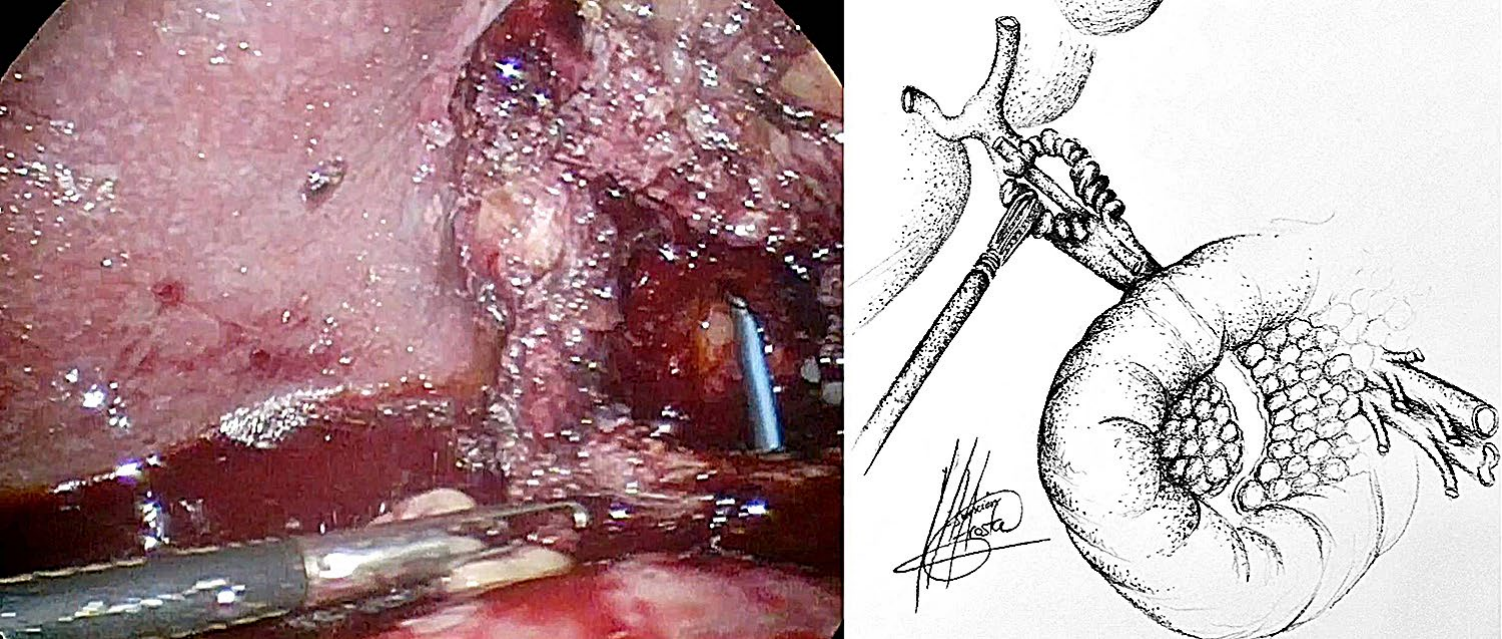
Stent in CBD

**Fig. 5:**
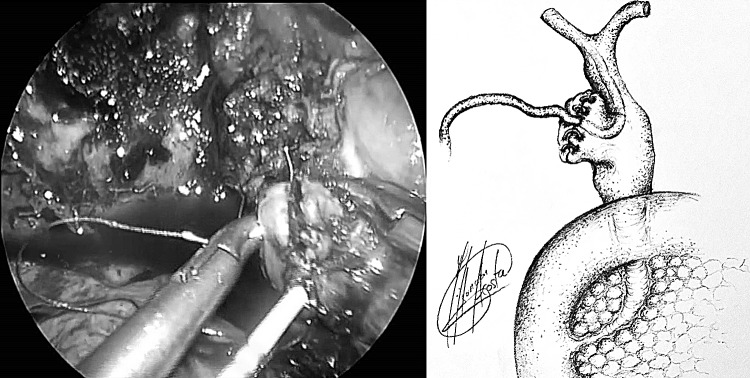
Closure of CBD over cholangiocatheter

Repair of the fistula and bile duct was performed over a T-tube (*n* = 3) (Fig. 3), a stent (*n* = 5) (Fig. 4) or a cholangiocatheter (*n* = 2) (Fig. 5). From the four patients that had pre-operative ERCP, stents were placed in three of them (cases 5, 7, 9). In two patients, the stent placed during ERCP was left in situ when repairing the CBD. In the remaining patient, the stent was removed and the CBD closed over a cholangiocatheter (case 7). The cholangiocatheter was used to perform a tubogram in order to assess the anatomy approximately 6 weeks after surgery. One patient underwent simple closure of the cystic duct with an Endoloop as the transinfundibular route was used (case 11). All patients except one had placement of an intra-abdominal drain in the liver bed (Table 2). No patients required conversion to open surgery.

Three patients developed post-operative complications: bile leak (*n* = 2, 18%) and lower respiratory chest infection (*n* = 1, 9%). All three complications were Clavien–Dindo grade 2 and managed conservatively. The International Study Group of Liver Surgery (ISGLS) published severity grading of bile leakage after hepatobiliary and pancreatic surgery [23]. The bile leaks from this case series were both graded B. Type II Mirizzi syndrome was associated with more bile leaks when compared to the rest of the series, however, this did not reach significance (18.2% vs 3.4%, *p* = 0.06). The median length of hospital stay was 5 days (Table 3). In one patient, histological examination revealed gallbladder carcinoma, which resulted in subsequent staged resection of the CBD and hepatectomy at the regional hepatopancreaticobiliary (HPB) centre.

**Table 3:** Outcome data

Case number	Complications	Clavien–Dindo classification	LoHS (days)	Readmissions
1	Bile leak	II	9	No
2	No		12	No
3	No		2	No
4	No		6	No
5	No		5	No
6	Bile leak	II	11	No
7	No		4	No
8	No		9	No
9	No		4	No
10	Chest infection	II	1	No
11	No		1	No

*LoHS* length of hospital stay

## Discussion

The true incidence of Mirizzi syndrome is unknown, as most of the cases are diagnosed in symptomatic patients undergoing biliary surgery, while asymptomatic cases may go undetected [3]. Most of the large patient series estimate the incidence of types I and II Mirizzi syndrome between 0.05 and 2.7% among patients with symptomatic gallstone disease in western developed countries [8, 24]. However, in Asia, Central and South America the incidence of Mirizzi syndrome is higher (4.7–5.7% of symptomatic gallstone disease) [6, 11, 12]. Mirizzi syndrome is more frequent in women than men with mean age of presentation 44–68 years (range 22–95 years) [4, 11, 17]. The incidence of type II Mirizzi syndrome is estimated in 0.1–0.5% of all patients submitted to surgery for symptomatic gallstones [3]. To the best of our knowledge, the incidence of type II Mirizzi syndrome in patients undergoing LCBDE for proven or suspected choledocholithiasis is not known. In our series of laparoscopic CBD exploration, the incidence of type II Mirizzi syndrome was 2.6%. The probability of an unsuspecting surgeon discovering type II Mirizzi syndrome intra-operatively during cholecystectomy is about 1 per 1250–2500 cases [3, 12]. In the UK, national guidelines advocate LCBDE at the time of laparoscopic cholecystectomy for the management of choledocholithiasis and concomitant gallstones [25]. As LCBDE becomes more widely adopted, surgeons should be aware that approximately 1 in 40 cases of LCBDE may result in uncovering type II Mirizzi syndrome intra-operatively.

There is an increased incidence of gallbladder cancer in Mirizzi syndrome compared with isolated cholelithiasis [7]. Gallbladder carcinoma has been associated with various degrees of Mirizzi syndrome [6]. Six to 24% of patients diagnosed pre-operatively with Mirizzi syndrome (mainly type II) are associated with gallbladder cancer. Patients with Mirizzi syndrome and biliary-enteric fistulas associated with gallbladder cancer have been reported. The main risk factors for gallbladder cancer are longstanding gallstone disease and stasis, which are also present in Mirizzi syndrome [5, 6, 13, 17]. There are no clinical features that help differentiate between a Mirizzi syndrome and a gallbladder cancer. Levels of CA 19-9 are highly elevated in gallbladder cancer (> 800 UI/mL), while they are only moderately elevated in benign disease associated with obstruction of the CBD. This could help to distinguish between them. When suspected pre-operatively, patients should undergo extensive studies including CT and MRCP [6]. One patient (9%) was diagnosed with gallbladder cancer within our series, who underwent further resection of the CBD and hepatectomy.

**Fig. 6:**
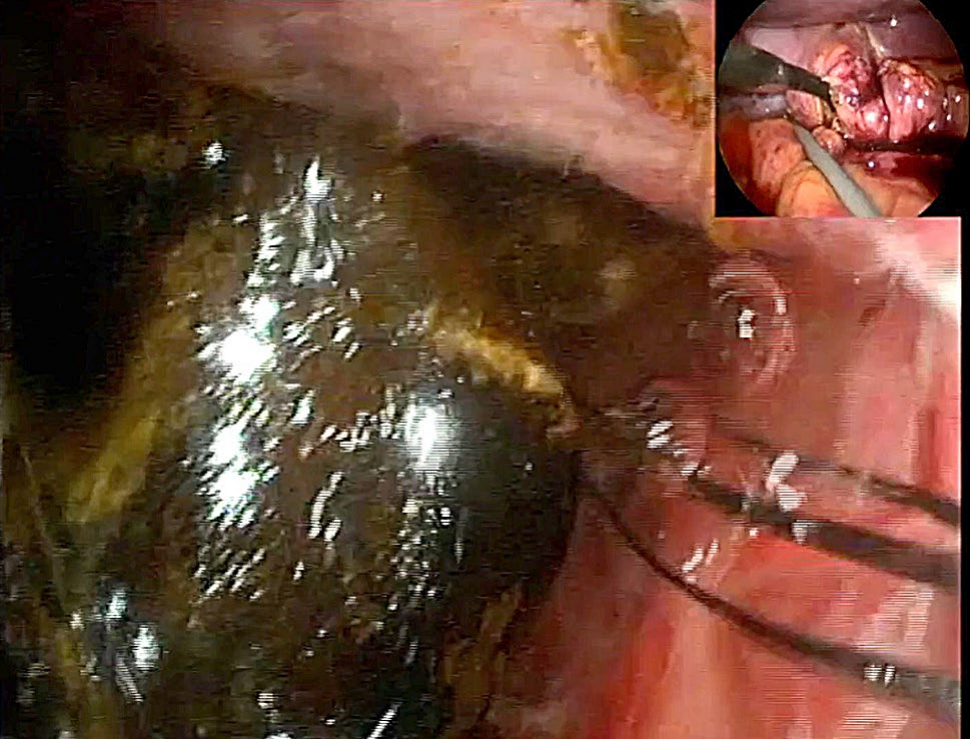
TIA access to CBD, stone impacted in CBD retrieved by choledochoscopy

Pre-operative diagnosis of Mirizzi syndrome is challenging because of the absence of pathognomonic signs and symptoms, and low sensitivity rates of imaging tests. It is only achieved in 8–62.5% of cases [6]. In our series the preoperative diagnosis rate was 27%. ERCP has the highest sensitivity for the diagnosis of Mirizzi syndrome, ranging between 55 and 90% [6, 8, 13, 26]. It is considered a gold standard diagnostic tool, as it defines the cause, level and extent of biliary obstruction, as well as ductal abnormalities, including fistula [14, 18]. In our series four patients underwent pre-operative ERCP, none of which were diagnostic of Mirizzi syndrome. This may have been due to incomplete assessment of the CBD as there was difficulty in cannulating, clearing or imaging the CBD. Ultrasonography is the most commonly used imaging procedure in the diagnosis of gallstones. The sensitivity of US for the diagnosis of Mirizzi syndrome ranges 8.3–62.7% [6, 8, 13, 26]. However, in our series, none of the pre-operative US scans performed yielded a positive diagnosis. MRCP is commonly used as it is non invasive and has a high sensitivity, similar to ERCP (50–90%) [6, 26]. MRCP also delineates the cause, level and extent of biliary obstruction, as well as ductal abnormalities, including fistula [14]. In our series MRCP was the pre-operative diagnostic tool with highest sensitivity (50%). CT scan is useful in differentiating Mirizzi syndrome from a malignancy, especially when a cholecystobiliary fistula is present [14]. Pre-operative diagnosis improves outcomes of surgery, allowing better planning [5, 6, 8, 27–29] and referral to the appropriate surgeon. If pre-operative diagnosis is not achieved, intra-operative recognition and proper management are essential to improve outcomes, reduce morbidity (up to 17% bile duct injury) and mortality [5, 13]. Furthermore, the intra-operative diagnosis of type II Mirizzi syndrome is challenging due to two main reasons. Firstly, Mirizzi syndrome implies longstanding chronically inflamed tissue that is difficult to manipulate and dissect. As a consequence, the gallbladder is fibrosed and adhered to the bile duct making visualization of the anatomy of the fistula and bile duct difficult [6]. Secondly, there is no standard measure to establish the difference between a dilated cystic duct and type II Mirizzi syndrome (as a consequence of longstanding impacted stones that erode into the CBD) if a transinfundibular approach of the fistula is chosen. Perhaps a dilated cystic duct is the initial stage of an evolving cholecysto-biliary fistula. This is demonstrated in our series by the last two cases (cases 10 and 11) where the stone was impacted within the CBD at the site of the junction with the cystic duct (Fig. 6). This is in comparison with the complete destruction of the CBD between the proximal and distal duct in case 8 which was repaired over a T-tube (Fig. 3).

**Fig. 7:**
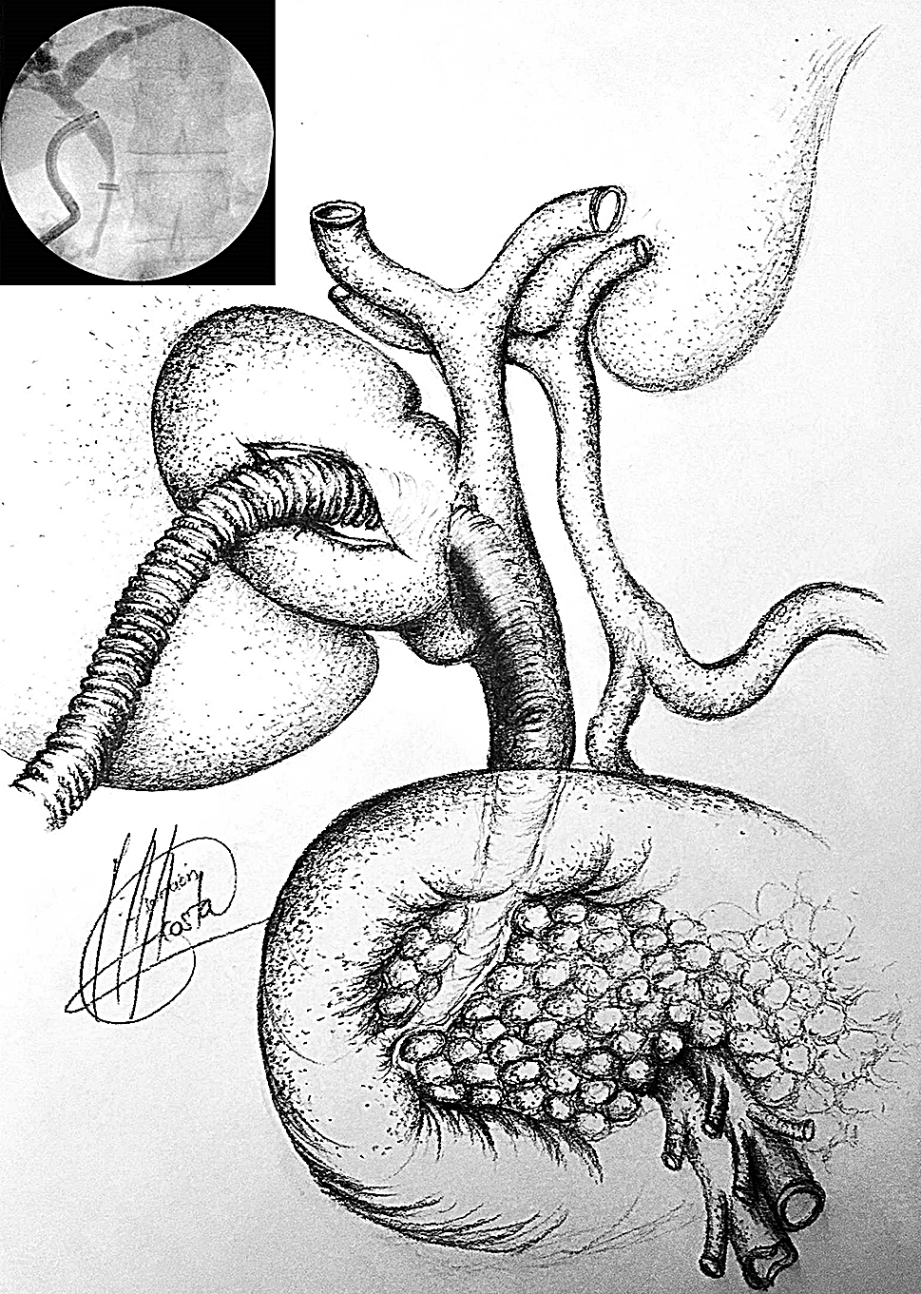
IOC case 11, access from infundibulum to CBD with choledochoscope through cholecystobiliary fistula

There is no standard treatment for Mirizzi syndrome. Treatment is often dependent on the available surgical expertise and intra-operative findings [5, 12, 29] but ultimately aims to reconstruct the anatomy by recruiting the infundibular part of the fistula. The traditional approach for type II Mirizzi syndrome included a subtotal cholecystectomy leaving a 10 mm flap of gallbladder around the fistula for the reconstruction of the destroyed bile duct protected by a T-Tube to avoid post-operative strictures or bile leakages. In cases with large defects (> 50% of the circumference of the CBD) when the surrounding tissues cannot be recruited to reconstruct the CBD bilioenteric anastomosis may be required [3, 5, 28] (Fig. 3). Moreover, some authors have reported successful treatment of complex bile duct stones in selected patients with and without Mirizzi syndrome using peroral cholangioscopy-directed lithotripsy. However, the rate of stone recurrence was high (16%) [13]. We described the TIA for accessing the bile duct in cases of a frozen hilum secondary to severe inflammation and/or fibrosis [12]. Chuang et al. described a similar approached to the bile duct but named it differently (transfistulous approach) [17]. As demonstrated by the last two cases in our series, we believe that a TIA or trans-infundibulo-fistulous approach (TIFA) augmented with the LABEL technique (when necessary) can avoid a fistulotomy or choledochotomy. Through an infundibular longitudinal incision (Fig. 1), this technique allows the removal of impacted gallstones and a more complete assessment of Hartmann’s pouch, the cystic duct area and the CBD. The reflux of bile indicates the presence of a fistula between the gallbladder/infundibulum/dilated cystic duct and the bile duct, as the cystic duct is usually occluded [5]. IOC and choledochoscopy will also help to identify the anomalous fistula anatomy and perform a safer dissection within the hepatic hilum, aided by the LABEL technique in cases with large stones [5, 19, 20, 22]. Since the introduction of the transinfundibular approach at our institution, we have noticed a decrease in the cases labelled as type II Mirizzi syndrome. However, there was no statistical difference in the incidence of type II Mirizzi syndrome in our series before and after implementation of TIA. Series of type II Mirizzi syndrome from Asian countries report a higher incidence when compared to our series: 11/425 in our series vs. 11/103 in Chuang et al. series [17]. Is this because the incidence of Mirizzi syndrome is higher in Asia or because patients within our series that had CBD exploration via TIA/transcystic route were labelled as dilated cystic ducts rather than Mirizzi syndrome [12]. Moreover, the difference between type II Mirizzi syndrome and a dilated cystic duct is not clearly defined when TIA is used as the impacted stones can be removed through the infundibulum. It is therefore not necessary to dissect the hilum, expose the fistula and perform a choledochotomy or fistulotomy when the procedure is completed by TIA. Impacted stones produce local inflammatory changes that distort the hilar and cystic duct anatomy. As a consequence, when exploring the CBD via TIA the choledochoscope directly accesses the stones lodged in the CBD without recognizing the anatomical features of the fistula/dilated cystic duct (Fig. 7). Due to this, intra-operative diagnosis of type II Mirizzi syndrome can be challenging as we experienced with the last two patients of the series. In our usual clinical practice, over 90% of patients undergo CBD exploration through the transcystic route, sometimes allowed by TIA when the dissection of the cystic duct is hazardous. Finally, when possible, a retrograde subtotal cholecystectomy is performed, and the closure of the CBD is performed with the flap of the infundibulum over a T-tube, a cholangiocatheter placed through the fistula or a stent to avoid post-operative bile leakages and strictures (Fig. 4). If patients were referred following preoperative ERCP with stent placement, closure of the CBD was performed over the pre-existing stent. In patients that did not undergo preoperative ERCP or a stent could not be placed during ERCP, the CBD was closed over a T-tube, a transcystic stent or a cholangiocatheter. The TIA approach has the advantage of not requiring choledochotomy or fistulotomy, thereby avoiding post-operative complications associated with bile leaks. However, in these cases we recommend leaving a transcystic drain to decompress the CBD which also allows post-operative cholangiography to assess that the anatomy has been restored [12]. An intra-abdominal drain is always left in the subhepatic region unless the infundibulum/dilated cystic duct can be closed with an Endoloop as we did in the last case.

Laparoscopy has traditionally not been recommended for the treatment of complex Mirizzi syndromes (McSherry type II) due to high risk of biliary injury (up to 22%) during dissection of the cystic duct and artery in an inflamed, distorted hilum and high conversion rates to open surgery (11.1–80%) [4–7, 13, 17, 18, 24, 27, 29–34]. Some groups advocate that laparoscopy is only feasible in type I Mirizzi syndrome [13, 14, 16]. In our experience laparoscopy is no longer a contraindication for the treatment of Mirizzi syndromes with a cholecystobiliary fistula. It is not strictly necessary to obtain a “critical view” of Calot’s triangle or perform a choledochotomy for exploring the CBD [5, 10, 18, 19, 24], avoiding hazardous dissection and further biliary tract injuries. There were no conversions to open surgery in our series. Two patients had a bile leak during the post-operative period that resolved with conservative management (Clavien–Dindo grade 2). We therefore advocate that a laparoscopic TIFA is a safe and feasible technique when performed by experienced surgeons. It provides the advantages of laparoscopy in terms of post-operative outcomes with reduced morbidity and shorter hospital stay whilst providing appropriate treatment for type II Mirizzi syndrome.

## Conclusions

The laparoscopic approach is safe and feasible for the treatment of type II Mirizzi syndrome. New approaches such as TIA and complementary techniques (e.g. LABEL) can be useful in the management of complex cases, avoiding choledochotomy or fistulotomy and their associated complications. Use of TIA may result in fewer cases of type II Mirizzi syndrome being identified intra-operatively and therefore accurate pre-operative diagnosis is important. Surgeons performing LCBDE for proven or suspected choledocholithiasis should be aware of the relatively high incidence of type II Mirizzi syndrome within this population and possess the necessary laparoscopic expertise.

## Electronic supplementary material

Below is the link to the electronic supplementary material.

Supplementary material 1 (MP4 276843 kb)
